# Experience of a Medical Expert

**Published:** 1886-10

**Authors:** F. A. Schmidt

**Affiliations:** Schulenburg, Texas


### THE EXPERIENCE OF A MEDICAL EXPERT.

*By F. A. Schmidt, M. D.*

[For Daniel's Texas Medical Journal.]

READING a paper on "Expert Testimony and Compensation" in a late Medical Journal, reminds me of my sad experience as an expert witness in a rape case, which happened several years ago, and which I here relate, for the purpose of contributing my share in bringing about a change in public sentiment and thereby, perhaps, acting upon our law-makers in initiating a new and better order of things.

An attempt had been made in my neighborhood—Austin County, my former home—to outrage the person of a little girl nine years old, on her way home from school. The supposed perpetrator had been "tracked," followed and arrested the same day, at the county seat, ten miles from the scene of action. About midnight of that day I was aroused by a deputy sheriff, and commanded to at once proceed to the house of the outraged child's parents, and there, with a professional brother of an adjoining settlement, to examine the injured child—for forensic purposes. I, of course, unhesitatingly obeyed the order, saddled my horse, prepared myself with the necessary armamentarium, and after an hour's ride, we arrived at the place of designation.

After having finished my task, my companion in adversity, Dr. E. and myself, were at once summoned by the officer to appear the next day at Bellville, the county seat, ten miles distant, as witnesses in a preliminary examination.

At Bellville we once more were commanded by the officiating Justice to examine the child's condition, which we did. The preliminary judicial proceedings lasted until night, and we two forcibly made witnesses were held all day at the county seat, of course at our expense, and to the detriment of our professional duties in our respective districts.

The supposed criminal was held to await the action of the grand jury, and after this body had indicted the supposed offender, we, forcibly made witnesses, were summoned to appear, as is customary in such proceedings.

This time two days were abstracted of our time, and the hotels being crowded, expenses running up quite high.

But the case was at last postponed. At the next term of court two days more of our time and the necessary expenses were incurred.

The jury in the case being "hung," it naturally came up at the next, but was, as customary, again postponed; our time and earnings, at these trials being squandered as on former occasions. In this way the case was manipulated over and over again.

Meanwhile I had removed to Schulenburg, in an adjoining county. Sometime after my removal, on arriving home from a long professional ride in the country, weary, worn out and indisposed, I was met at the gate by an officer of the law who presented "attachment" for the body of one F. A. Schmidt. I was formally arrested, and in company of a sheriff summarily put on board of the midnight train and transported to Bellville—being 150 miles distant by rail—where again court was in session, and where probably my case would be acted upon.

The case had, however, already been postponed, and I was permitted to return home, but not before "his honor," the District Judge, had cited me before him in open court, and had placed me under heavy penalty—$500 bond—to insure my next appearance, and, in order, as "his honor" remarked, to save the State the cost of attachment; leaving me, poor victim, suffering for having spent time, money and assiduity in learning a profession which entitled the State to deal with me as though it was a felony to study and I a criminal who deserved punishment. Being thus under heavy bond, it became necessary on my part to watch the case and holding myself in readiness to appear, at a moment's notice, perhaps. Not by any means. I had been summoned three years before, and our statutes do not require a second notice of the re-convening of the court. I had to keep myself informed as best I could, and the expensive telegraph had to be resorted to by me, so as to receive timely notice of my required presence. In short, I was at the entire pleasure of the judge and the lawyers concerned in the case, from beginning to end.

The case was at last dismissed, and your humble servant minus—not to count loss in practice, worry, and anxiety—over two hundred dollars cash outlay, for which I received no remuneration whatever, my "expert" testimony thrown in.

Such has been my experience as an expert witness, forcibly made such by command of officers of the law. I could recount a number of similar experiences of other professional brethren, and I see no reason why they should not happen to this day, for I have it on good authority that all this is perfectly legal. Is it, then, to be wondered at that I, and other victims of the blindfolded goddess, shun expert cases ever since, and *that the State* loses our services, and justice our testimony, *whenever we can devise means*, proper or improper—at any rate demoralizing—to defeat the courts in trespassing on our time, knowledge, purse, and, what is worse, on our personal liberty? Ought such intolerable condition to continue? Should the State, as it certainly does, contribute in demoralizing its best citizens by forcing them, for self-protection, to become of *loose veracity* and *reserved in the fulfillment of their humanitarian duties?* I should think not, and the sooner our law-makers change this condi tion of things, the better for all concerned.

Schulenberg, Texas.

